# Enhanced lower-limb motor imagery by kinesthetic illusion

**DOI:** 10.3389/fnins.2023.1077479

**Published:** 2023-06-20

**Authors:** Weizhen Wang, Bin Shi, Dong Wang, Jing Wang, Gang Liu

**Affiliations:** ^1^Institute of Robotics and Intelligent Systems, School of Mechanical Engineering, Xi’an Jiaotong University, Xi’an, China; ^2^Henan Key Laboratory of Brain Science and Brain-Computer Interface Technology, School of Electrical Engineering, Zhengzhou University, Zhengzhou, China

**Keywords:** brain-computer interface, lower-limb, motor imagery, kinesthetic illusion, vibratory stimulation, electroencephalogram

## Abstract

Brain-computer interface (BCI) based on lower-limb motor imagery (LMI) enables hemiplegic patients to stand and walk independently. However, LMI ability is usually poor for BCI-illiterate (e.g., some stroke patients), limiting BCI performance. This study proposed a novel LMI-BCI paradigm with kinesthetic illusion(KI) induced by vibratory stimulation on Achilles tendon to enhance LMI ability. Sixteen healthy subjects were recruited to carry out two research contents: (1) To verify the feasibility of induced KI by vibrating Achilles tendon and analyze the EEG features produced by KI, research 1 compared the subjective feeling and brain activity of participants during rest task with and without vibratory stimulation (V-rest, rest). (2) Research 2 compared the LMI-BCI performance with and without KI (KI-LMI, no-LMI) to explore whether KI enhances LMI ability. The analysis methods of both experiments included classification accuracy (V-rest vs. rest, no-LMI vs. rest, KI-LMI vs. rest, KI-LMI vs. V-rest), time-domain features, oral questionnaire, statistic analysis and brain functional connectivity analysis. Research 1 verified that induced KI by vibrating Achilles tendon might be feasible, and provided a theoretical basis for applying KI to LMI-BCI paradigm, evidenced by oral questionnaire (Q1) and the independent effect of vibratory stimulation during rest task. The results of research 2 that KI enhanced mesial cortex activation and induced more intensive EEG features, evidenced by ERD power, topographical distribution, oral questionnaire (Q2 and Q3), and brain functional connectivity map. Additionally, the KI increased the offline accuracy of no-LMI/rest task by 6.88 to 82.19% (*p* < 0.001). The simulated online accuracy was also improved for most subjects (average accuracy for all subjects: 77.23% > 75.31%, and average F1_score for all subjects: 76.4% > 74.3%). The LMI-BCI paradigm of this study provides a novel approach to enhance LMI ability and accelerates the practical applications of the LMI-BCI system.

## 1. Introduction

Stroke disables or kills several million people each year (Beal, 2010; Katan and Luft, 2018). The disability, especially the lower limb hemiplegia, highly impacts the lives of individuals (Rea et al., 2014; Katan and Luft, 2018). Rehabilitation therapy is vital for helping the survivor regain as much use of his/her lower limbs as possible. Traditional therapy acts on the distal physical level to indirectly influence the brain’s neural system, such as physical therapy and occupational therapy (Belda-Lois et al., 2011). However, these indirect therapies usually have poor efficiency. BCI directly detects and modulates brain activity (Abiri et al., 2019; Saha et al., 2021). There are two main BCI strategies to improve the lives of individuals among stroke patients, i.e., assistive BCI and rehabilitative BCI (Mane et al., 2020). In the last decade, rehabilitative BCI has emerged as one of the promising tools for lower-limb motor function restoration by adjusting neuronal plasticity in affected neural circuits (Mane et al., 2020; Romero-Laiseca et al., 2020; Bobrova et al., 2021). In the field of BCI, a lower limb exoskeleton control system based on steady state visual evoked potentials (SSVEP) is an efficient BCI system, such as achieved accuracies of 91.3 ± 5.73% and an information transfer rate (ITR) of 32.9 ± 9.13 bits/min (Kwak et al., 2015). However, recent study reviewed that the flickers used to encode the BCI command must be sufficiently intense to obtain a high-quality SSVEP, which will engage a relatively large portion of the visual resources. Moreover, such irritating visual stimuli are not only irrelevant to users’ subjective intent, but even disrupt users and make them feel uncomfortable. Therefore, even though this BCI system can work well to assist subjects with walking, its unnatural way of interaction is unacceptable to some users, which reduces its usefulness in practice (Xu et al., 2021).

LMI-BCI is a rehabilitative BCI and is natural. It detects the decrease/increase of power in the sensorimotor cortex to control external equipment and then reversely modulates brain activity by external equipment (Pfurtscheller and da Silva, 1999; Abiri et al., 2019; Zhang et al., 2021). Two types of MI can be distinguished: Kinesthetic Motor Imageries (KMI) and Visual Motor Imageries (VMI). A KMI can be described as the ability to imagine performing a movement without executing it, by imagining haptic sensations felt during the real movement (i.e., tactile, proprioceptive, and kinesthetic). In comparison, VMI represents a visualization of the corresponding movement incorporating the visual network (Rimbert et al., 2018; Yang et al., 2021). Nevertheless, there are three challenges for MI-BCI system: (1) EEG features is unstable in general; (2) It is difficult to detect lower-limb EEG features because the anatomical location of the lower-limb motor cortical area deep within the contralateral mesial cortex (Neuper and Pfurtscheller, 1996; Pfurtscheller et al., 1997; Pfurtscheller and da Silva, 1999; Boord et al., 2010); (3) Brain injury often inhibits the LMI ability after stroke, making the detection of EEG features harder (Takeda et al., 2007; Meyer et al., 2016; Park et al., 2016).To address the above challenges, several studies focused on enhancing LMI ability by Mirror Neuron system (MNS). MNS can transform visual sensory input of related behavior (e.g., Action Observation, AO) into one’s own brain impulse or motor output of behavior (Kohler et al., 2002; Rizzolatti and Sinigaglia, 2016; Tanaka, 2021). Li et al. (2015) enhanced LMI ability in an imagining playing football task by Action Observation and demonstrated more distinctive features in LMI with AO than without. As a kind of action observation, virtual reality (VR) plays an essential role in enhancing neural activity during motor imagery. These studies suggest that the use of immersive virtual reality headsets, with the illusion and embodiment they provide, can effectively improve motor imagery training and BCI performance (Choi et al., 2020; Ferrero et al., 2021). In the field of lower-limb rehabilitation, standing and sitting are two regular movements. The experimental results of Triana-Guzman et al. indicated that the classification of motor imagery and idle state provided a mean accuracy of 88.51 ± 1.43% and 85.29 ± 1.83% for the sit-to-stand and stand-to-sit transitions, respectively (Triana-Guzman et al., 2022). Additionally, Chaisaen et al. (2020) classified the AO/MI of sit-to-stand/stand-to-sit task, and the highest mean accuracy is 82.73 ± 2.54%.

In 2021, a latest study revealed that mimicking known biological control principles results in BCI performance that is closer to healthy human abilities (Flesher et al., 2021). Brain-muscle-kinesthesis loop is the known biological control principle (Daly and Wolpaw, 2008). Hemiplegia inhibits kinesthesis after stroke(De Vries and Mulder, 2007; Mane et al., 2020). kinesthetic illusion will complement the relevant loop. Neuroimaging studies have revealed that a relationship exists between the movement that has been imagined and the activation patterns of somatotopically organized motor and kinesthetic areas (Giannopulu and Mizutani, 2021). In the upper-limb MI-BCI study, KI induced by rubber hand can significantly amplify EEG features and provide better guidance to enhance upper-limb MI (Song and Kim, 2019). No previous study has investigated enhancing LMI-BCI performance by KI, although KI has important effects on BCI. This paper proposed an enhanced LMI paradigm by KI. Specifically, earlier studies proved that kinesthetic illusion where one feels muscle stretch could be induced by artificially vibrating the muscle spindle and tendon (Goodwin et al., 1972; Naito and Ehrsson, 2001; Naito, 2004; Tapin et al., 2021). Therefore, we induced KI by vibratory stimulation on Achilles tendon to enhance LMI ability during imagining kicking a football. We designed both research contents. The first research verified the feasibility of induced KI by vibratory stimulation and compared the brain activity during V-rest and rest task. The second research explored the effect of KI on LMI by comparing the LMI with and without KI (KI-LMI vs. no-LMI).

The rest of this paper is organized as follows. The experimental description and analysis methods are introduced in Section 2. Then, results are illustrated in Section 3 in terms of EEG analysis and BCI performance. Finally, discussion and conclusion are presented in Sections 4 and 5, respectively.

## 2. Materials and methods

### 2.1. Participants

Sixteen healthy subjects without a history of any neurological diseases (Subject H1-H16, all right-handed, age: 25 ± 2.98 years) participated in this study. Participants were asked to sleep normally and refrain from alcohol, caffeine and stimulant foods for 24 h before the experiment. All subjects were informed about the experimental process and required to sign an approved informed consent form before participating. This study was approved by the Ethics Committee of Xi’an Jiaotong University (Approval No. 2021–1,577).

### 2.2. Required equipment

**Figure 1:**
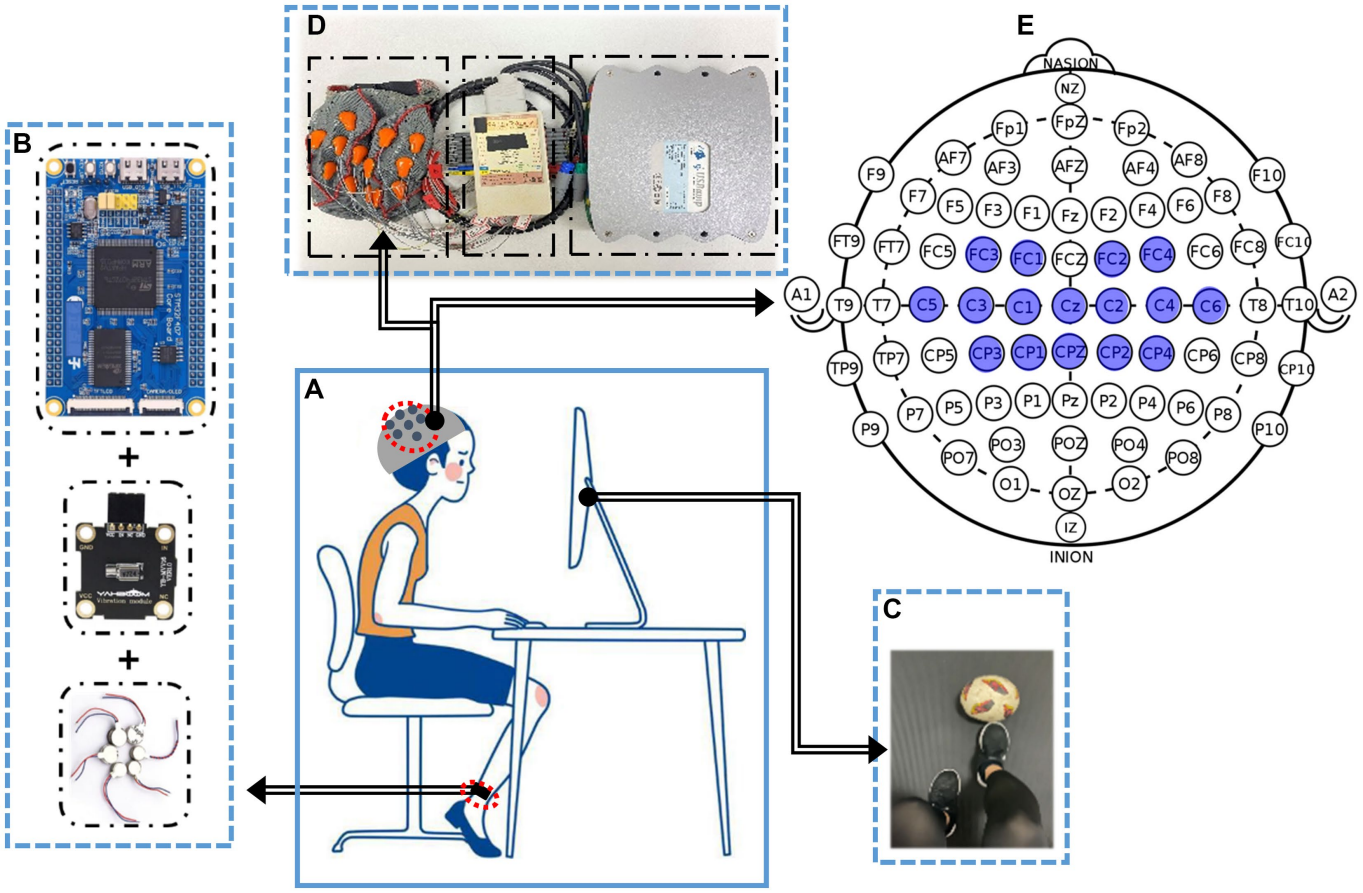
The experimental framework of enhanced LMI by KI. **(A)** Experiment condition, i.e., subjects were seated in a comfortable chair with feet resting on the floor in front of the monitor. **(B)** A inducing Illusion device. Kinesthetic illusion was induced by artificially vibrating the Achilles tendon. **(C)** Visually induced interative interface. **(D)** EEG acquisition equipment. **(E)** Detailed channel locations are placed in the sensorimotor cortex (i.e., blue marked area).

Previous researches have revealed that activated cortex area (i.e., contralateral primary motor cortex) by KI was similar to the activated cortex area by MI (Naito, 2004; Lopez et al., 2017; Giannopulu and Mizutani, 2021). Therefore, EEG signal was recorded using 16-channel active electrodes placed over the sensorimotor cortex (Detailed locations are illustrated in Figure 1E), with the g.USBamp (g.tec Inc., Austria) system (See Figure 1D for the composition structure) according to the 10–10 electrode location system (Jurcak et al., 2007). The reference electrode and the ground electrode were placed on A1 and A2, with the sampling rate of 1,200 Hz. To reduce artifacts and power line interference, impedances for all electrodes were kept below 5 KΩ. Meanwhile, an online band-pass filter between 0 and 60 Hz and a notch filter between 48 and 52 Hz were applied on the raw EEG. During the task, subjects were sat in a comfortable chair with feet resting on the floor in front of the monitor as shown in Figure 1A.

In terms of visual induction, the Psychtoolbox-3 toolbox is utilized to design a visual induction interface (i.e., Figure 1C). In the aspect of inducing illusion device, the microcontroller (STM32F4) sends Pulse Width Modulation (PWM) regulation signals to a vibration regulation module, and then the eccentric vibrator (RswTech-motor-0827) generates specified frequency. Detailed structure is shown in Figure 1B. The vibration stimulation device in this study has a vibration frequency of 180 Hz under rated voltage. Additionally, Pacinian corpuscles in the mechanical receptor of human skin are sensitive to above 100 Hz frequency (Breitwieser et al., 2012). Therefore, a vibration frequency of 180 Hz was selected for experimental investigation in this study.

### 2.3. Data acquisition

**Figure 2:**
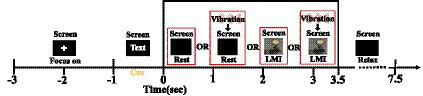
Overview of the different task paradigms. The process included four different tasks, i.e., rest, V-rest, noLMI or KI-LMI.

Before the experiment, subjects were told to perform the LMI task of right limb. In addition, A inducing Illusion device was placed at the right Achilles tendon to induce kinesthetic illusion in right limb (see Figure 1A). Before the EEG acquisition, each participant needs to undergo 5 min training session by motor execution to become familiar with the experimental task. In this study, the pre-training method was used to make the subjects better actively to complete the LMI task. During the data acquisition, all subjects were required to avoid actual movements for collecting high-quality EEG data. All trials for participants were completed in 1 day to reduce the EEG variability in different time periods. This study collected EEG data under four different conditions. Detailed paradigms flow is described in Figure 2: At the beginning (*t* = −3 - -1 s) of each trial, a white cross was displayed on the center of the screen to remind subjects to stay focused. Subsequently, a text cue (rest, V-rest, no-LMI, or KI-LMI) appeared for 1 s. When ‘V-rest or rest’ is observed, only the black background was shown to the subject who was executing rest task with or without vibratory stimulation synchronously (i.e., *t* = 0–3.5 s). When ‘KI- LMI or no-LMI’ is observed, the designed visual guidance of kicking a football was shown to the subject who was executing LMI task with or without vibratory stimulation synchronously (i.e., t = 0–3.5 s). There was a 4 s relaxation at the end of each trial. According to the random text cue, each subject randomly implemented the rest/LMI task (see Figure 2) in order to exclude the effect of task order on the results. In order to reduce the fatigue of the subjects, this study take a 3 min break after each run. Each subject performed a total of 12 runs, and each run consisted of 8 trials for rest task and 8 trials for LMI task. Thus, a total of 192 trials (i.e., 8 × 2 × 12 = 192) were performed by each subject.

### 2.4. Research contents architecture

These collected data was applied to explore two research contents:

Research 1: This research aims to verify the feasibility of induced KI by vibrating Achilles tendon and analyze the EEG features produced by KI. Its process is as follows: (1) Oral questionnaire (Question1: Do you feel your feet moving during V-rest?) (2) The topographical distribution of rest and V-rest were compared to explore the independent effect of KI on cerebral cortex. (3) The EEG data of rest and V-rest were classified to quantify the independent effect of KI on rest task.

Research 2: This research aims to explore whether KI enhances LMI. Its process is as follows: (1) Topographical distribution, brain functional connectivity map and the ERD of Cz electrode (Cz-ERD) were analyzed during no-LMI and KI-LMI task. (2) The EEG data of rest and no-LMI/KI-LMI task were classified to explore the effect of KI on enhancing LMI. (3) Oral questionnaire (Question 2: Is KI conducive to focusing on LMI? Question 3: Which LMI do you prefer?) (4) Statistical test of C3/C4-ERD power explored whether KI conducive to distinguishing between left and right LMI.

### 2.5. Data analysis methods

#### 2.5.1. Event related desynchronization analysis

This study selected 1 s EEG data as the baseline from the cross period (i.e., $t_{\mathrm{base}}$ = −2.5 ~ −1.5 s, see Figure 2), since the subjects were in the most relaxed state (i.e., the signal was the most stable) during $t_{\mathrm{base}}$ . Additionally, it selected 3 s EEG data as the task segment from the task period (i.e., $t_{\mathrm{task}}$ = 0.5 ~ 3.5 s), since severe artifacts were found within 0 ~ 0.5 s. And, EEG data of the task period was used for preprocessing, feature extraction, and task classification. In order to enhance the quality of EEG data, all trials were visually inspected to remove data with more artifacts. The following exclusion criteria were applied to identify and discard noisy trials: (1) Maximum peak-to-peak value greater than 200 μV; (2) the subject is blinking, the electrodes are not making good contact with the scalp, or there are some muscle artifacts (Delijorge et al., 2020; Triana-Guzman et al., 2022). Finally, any epoch where at least one electrode met these criteria was visually inspected to rule out noise-contaminated trials and labeled as an “artifact” manually.

Fourty trails of each task were randomly selected from the remaining data for each subjects. Event Related Desynchronization (ERD) is caused the decrease of EEG frequency power in alpha (8-13 Hz) and beta (14-30 Hz) bands on the motor area related to the body parts by preparing movement. In contrast, Event Related Synchronization (ERS) is caused the increase of EEG frequency power in a similar way and bands (Machida and Tanaka, 2018). ERD power is one of the most common time-domain analysis methods for studying cerebral cortex activity during motor imagery. Therefore, we calculated the instantaneous power within a 0.25 s moving time window to describe the ERD change in the time domain (Phon-Amnuaisuk, 2008; Nakayashiki et al., 2014). In previous study (Pfurtscheller and da Silva, 1999; Graimann et al., 2002; Hashimoto and Ushiba, 2013), the classical method to compute the time course of ERD includes the following steps: (1) bandpass filtering of all event-related trials; (2) squaring of the amplitude samples to obtain power samples; (3) averaging of power samples across all trials; (4) averaging over time samples to smooth the data and reduce the variability. Similarly, the method is illustrated by equations (1–4) and applied to our research.


(1) $$\;P_{\mathrm{base}}=\frac{1}{T_{\mathrm{base}}}\underset{t\in T_{\mathrm{base}}}{\sum }P_{t}$$

(2) $$\begin{array}{c} \;P_{\mathrm{task}}=\frac{1}{T_{\mathrm{task}}}\underset{t\in T_{\mathrm{task}}}{\sum }P_{t} \end{array}$$

(3) $$\begin{array}{c} \;\mathrm{ERD}\left(t\right)=\frac{P_{t}-P_{\mathrm{base}}}{P_{\mathrm{base}}}\times 100 \end{array}$$

(4) $$\begin{array}{c} \;\bar{\mathrm{ERD}}=\frac{P_{\mathrm{task}}-P_{\mathrm{base}}}{P_{\mathrm{base}}}\times 100 \end{array}$$

Where, $\;P_{t}$ represents the instantaneous EEG power; $P_{\mathrm{base}}$ and $P_{\mathrm{task}}$ represent the average power during the period of $T_{\mathrm{base}}$ and $T_{\mathrm{task}}$ , respectively; ERD(t) represents instantaneous ERD; $\bar{\mathrm{ERD}}$ represents the average ERD of the task segment. Optimal time period of motor imagery were different due to the variability among subjects or trials. Furthermore, the $\bar{\mathrm{ERD}}$ of each channel was used to draw topographical distribution and execute statistical test.

#### 2.5.2. Functional connectivity analysis

To compare the patterns of two LMI tasks, we analyzed brain connectivity using the imaginary part of coherence (iCOH) algorithm. This algorithm is insensitive to artefactual caused by volume conduction, because a signal is not time-lagged to itself and thus manages to identify the synchronizations of two signals that are time-lagged (Nolte et al., 2004; Pezoulas et al., 2018). It is defined as:


(5) $$\begin{array}{c} \;\mathrm{iCOH}=\mathrm{imagin}\left(R_{x,y}\left(f\right)\right) \end{array}$$

where $\;R_{x,y}\left(f\right)$ is the coherence of signals *x*, *y*, at *f*. Coherence is defined as the absolute value of coherency. The latter measures the linear relationship of the two signals at *f*. In fact, coherence acts as a generalization of correlation to the frequency domain with its values varying on the interval [0, 1], where 1 indicates a perfect linear prediction of *y* from *x*.

**Figure 3:**
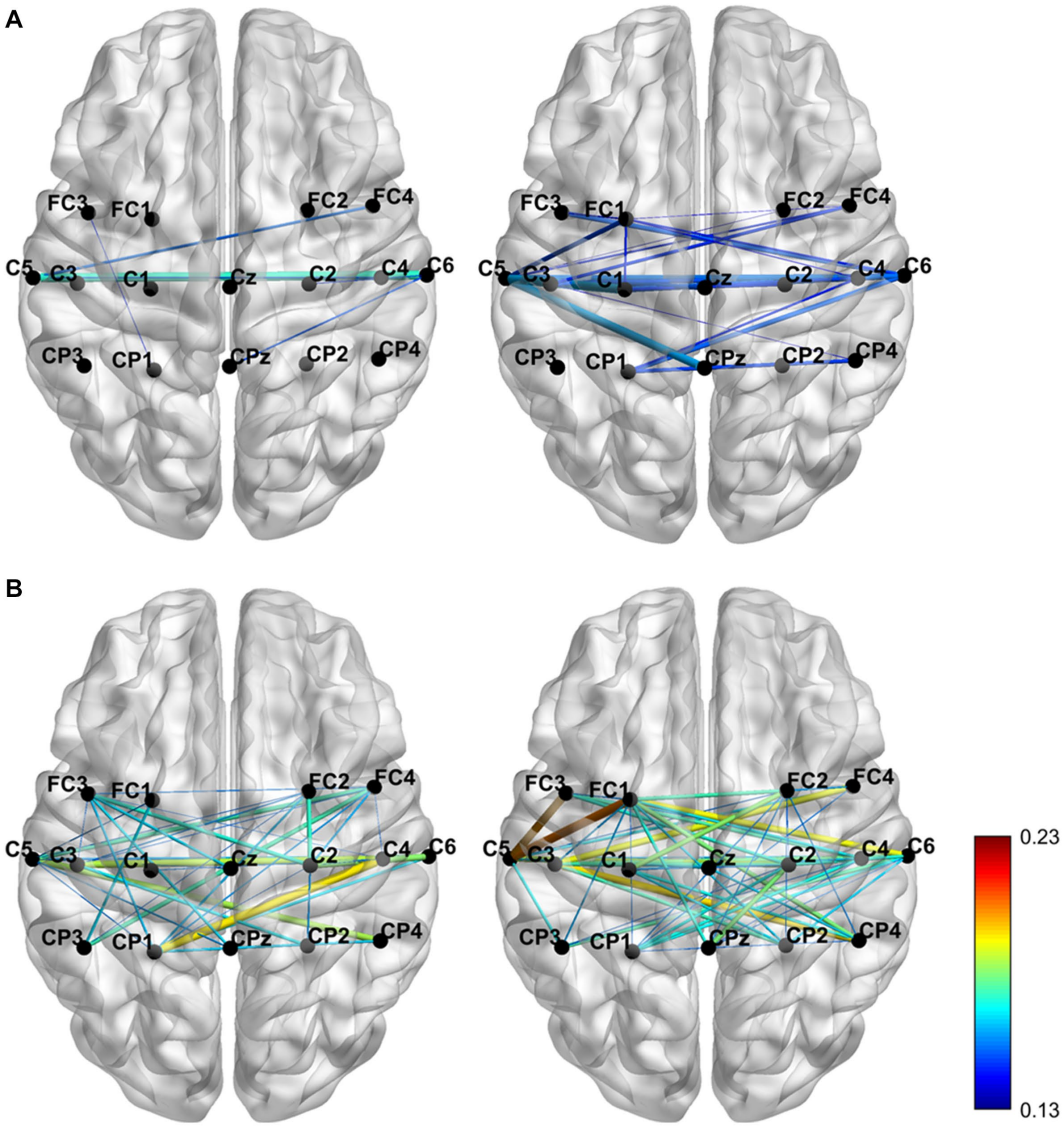
The brain functional connectivity map was grand averaged across subjects for the two conditions with the alpha **(A)** and beta **(B)** frequency bands separately.

In this study, the region of interest (ROIs) consists of 16 channels (Detailed channel locations are shown in Figure 1E). And, our study used iCOH algorithm between the signals at the paired cortical ROIs, and plotted the brain functional connectivity map (see Figure 3).

#### 2.5.3. Feature extraction and classification algorithm

**Figure 4:**
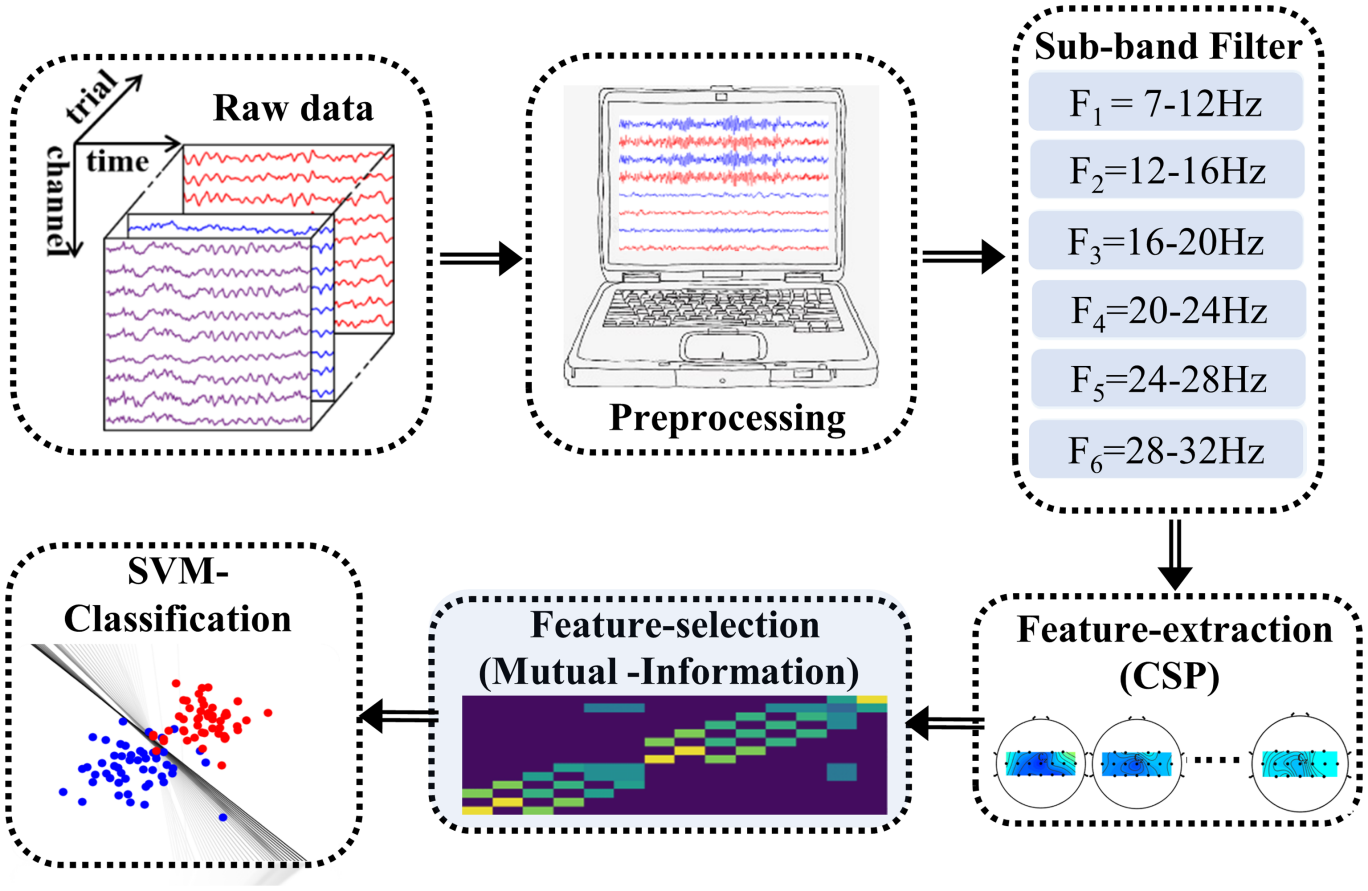
The Offline analysis process. This process includes the preprocessing, sub-band filter feature extraction (i.e., CSP), feature selection and classification.

Common Spatial Patterns (CSP) is the well-known feature extraction method for analyzing EEG signals. However, its classification result lies on a certain frequency range. In fact, the optimal frequency band of motor imagery is different among subjects (Li et al., 2015; Liu et al., 2019; Shu et al., 2019). In recent years, an improved feature extraction algorithm called Filter Bank Common Spatial Pattern (FBCSP) has been applied to solve this deficiency. This method includes three stages (see Figure 4): Firstly, the target data is divided into different frequency bands by a band-pass filter. Secondly, the CSP algorithm is used to extract features of all sub-band data automatically. Lastly, the optimal feature selection is performed based on mutual information theory (Ang et al., 2008, 2012). In this paper, we divided 7-32HZ frequency band into six sub-bands (i.e., 7–12, 12–16, 16–20, 20–24, 24–28, and 28-32 Hz). Finally, eight optimal target feature vectors are selected to achieve a better classification performance and reduce the information redundancy.

Support Vector Machine (SVM) with Gaussian kernel is suitable for classing the 2-class small samples of LMI (Yuan and Wang, 2008; Somadder and Saha, 2021). The Python platform is utilized to build a suitable SVM model. The optimal parameters have a vital influence on classification performance. Thus, a cross-validation method is used to optimize the parameter C in the cost function and the parameter γ in the radial basis function.

### 2.6. Evaluation

This study used accuracy (Acc) and F1_score to evaluate classification performance and stability of the classification model. The process of how to calculate Acc and F1_score is as follows (Ren et al., 2020):


(6) $$\begin{array}{c} \;\mathrm{Acc}=\frac{\mathrm{TP}+\mathrm{TN}}{\mathrm{TP}+\mathrm{TN}+\mathrm{FP}+\mathrm{FN}} \end{array}$$

(7) $$\begin{array}{c} \;F1\_\mathrm{score}=\frac{2.\frac{\mathrm{TP}}{\mathrm{TP}+\mathrm{FP}}.\frac{\mathrm{TP}}{\mathrm{TP}+\mathrm{FN}}}{\frac{\mathrm{TP}}{\mathrm{TP}+\mathrm{FP}}+\frac{\mathrm{TP}}{\mathrm{TP}+\mathrm{FN}}} \end{array}$$

Where, TP, TN, FN, and FP represent the relationship between the true-value and predicted-value (see Table 1).

**Table 1:** The confusion matrix of true-value and predicted-value.

Confusion matrix		True-value	
		1	0
Predicted-value	1	TP	FP
	0	FN	TN

In this study, we constructed four datasets (Dataset 1: Data of 40 rest trials and 40 V-rest trials; Dataset 2: Data of 40 rest trials and 40 no-LMI trials; Dataset 3: Data of 40 rest trials and 40 KI-LMI trials; Dataset 4: Data of 40 V-rest trials and 40 KI-LMI trials). Each data set is classified into two categories. Five-fold cross-validation is used to calculate the average accuracy.

The normality test results show that the significance level of the normality test is *p* > 0.05. EEG data fit a normality distribution. Therefore, statistical results of this paper were analyzed by paired T-test and One Sample T-test. These statistical test methods were calculated by using SPSS 24.0 mathematical tool. Subsequently, statistical graphs were drawn by MATLAB 2016 and GraphPad Prism 8.

## 3. Results

### 3.1. Research 1: verifying that KI is induced by vibrating Achilles tendon

This research aims to verify the feasibility of inducing KI *via* vibrating Achilles tendon and analyze the EEG features produced by KI.

#### 3.1.1. Oral questionnaire (Q1)

After completing all the experimental tasks, each subject was given an oral questionnaire (Q1) to explore the correlation between vibration stimulation and KI. Its results are shown in Table 2. Firstly, we inquired Q1 that do you feel your feet moving slightly during V-rest. Thirteen subjects’ answer is ‘Yes’. Three subject’s answer is ‘No’. In practice, however, their feet were not moving. Therefore, experiment 1 verified that KI (i.e., feel their feet moving) was felt subjectively by most subjects during vibrating Achilles tendon.

**Table 2:** The results of oral questionnaire.

Question	Content	Result (number)	
Q1	Do you feel your feet moving slightly during V-rest?	No	Yes
		3	13
Q2	Is KI conducive to focusing on LMI task?	No	Yes
		4	12
Q3	Which LMI paradigm do you prefer?	no-LMI	KI-LMI
		4	12

#### 3.1.2. Topographical distribution of rest and V-rest

**Figure 5:**
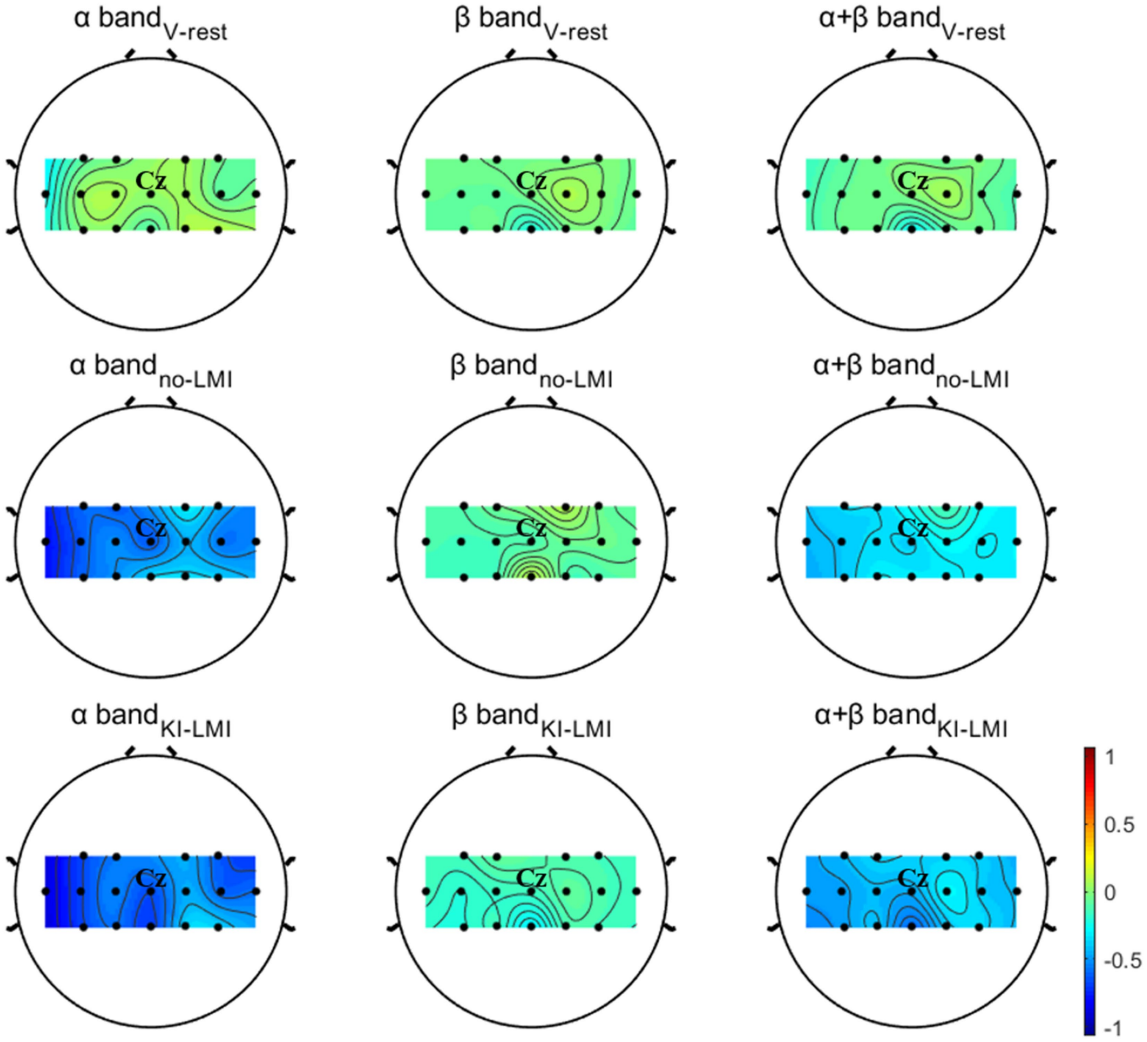
Grand-averaged topographical distribution for V-rest, no-LMI and KI-LMI task relative to the rest task. The color bar denotes ERD/ERS. The Cz represents the anatomical location of the lower-limb motor cortical area.

The first row of Figure 5 displays the grand-averaged topographical distribution for V-rest task relative to the rest task. In the period of V-rest task, there is a slight ERD phenomenon for Contralateral cerebral cortex (i.e., left hemispheric region) in both the β band and the α + β band, and the area around the Cz and CPz electrode (i.e., around the sensorimotor cortex) is predominantly activated. Therefore, induced KI by vibratory stimulation could not only be felt subjectively by subjects but also reflected in electrophysiological features.

#### 3.1.3. Quantifying the independent effect of KI on rest task

**Figure 6:**
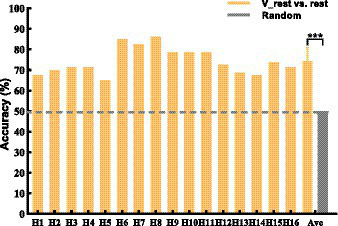
2-class accuracy of rest and V-rest. The black dashed line represents random (50%) of accuracy that means no difference between V-rest and rest. Data: mean ± SD. ***p < 0.001.

The brain topographic maps of V-rest and rest show differences (see Figure 5). Our study classified V-rest and rest to quantify the independent effect of KI on rest task. Classification performance of rest and V-rest for all subjects is shown in Figure 6. Results were statistically evaluated using a one-sample T-test. The lowest and highest accuracy are 65.0 and 86.25%, respectively. The average accuracy of all subjects achieved 74.3%, significantly higher than the random accuracy (50%). Therefore, there were statistically significant EEG features which is generated by KI.

### 3.2. Research 2: exploring whether KI enhances LMI

#### 3.2.1. Topographical distribution and Cz-ERD power of no-LMI and KI-LMI

The second and third rows of Figure 5 displays the grand-averaged topographical distribution for no-LMI and KI-LMI task relative to the rest task. In the period of no-LMI task, the significant activation region mainly occurred around the Cz electrode (i.e., around the sensorimotor cortex) in both the α band and the α + β band. Compared with the no-LMI task, the KI-LMI task can generate more obvious cortex activation at all frequency bands (i.e., significant ERD). And, there is most significant activation for the around Cz and CPz electrodes at all frequency bands. Especially, this activation is more localized to the contralateral side of the cerebral cortex during performing the KI-LMI task.

**Figure 7:**
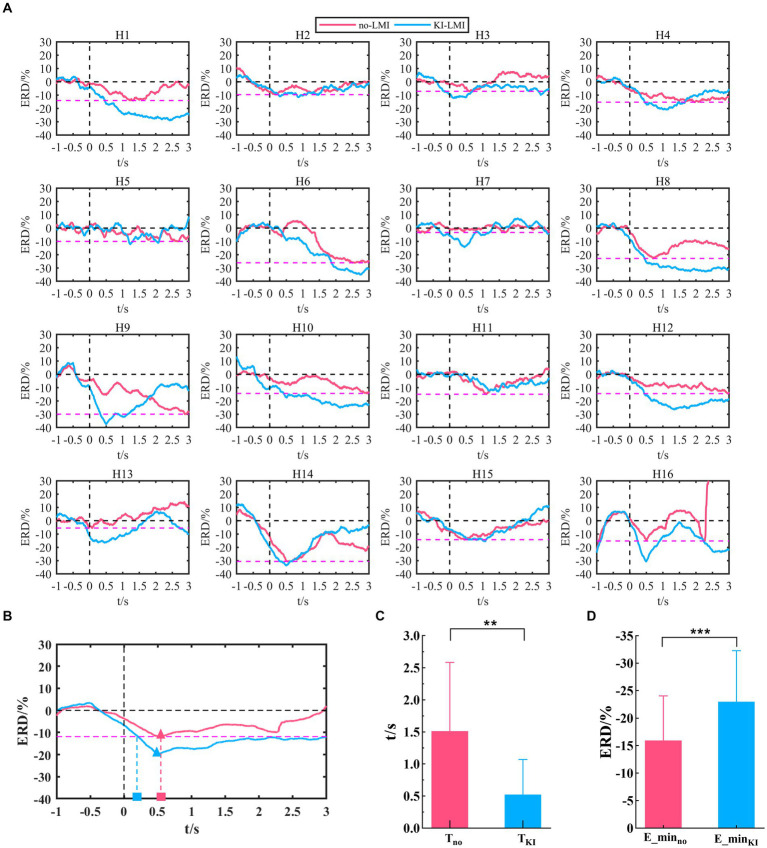
Cz-ERD power during LMI in the 8–30 Hz band. **(A)** Cz-ERD power of the 16 subjects. The black vertical dashed line indicates the start of LMI. The black horizontal dashed line represents the baseline. The red dashed line represents the lowest ERD power (E_min^i^_no_, i = 1, 2, 12) during the no-LMI task for each subject i. **(B)** Average ERD of all subjects for no-LMI and KI-LMI tasks. The red/blue triangle represents the lowest ERD power (E_min_no_ or E_min_KI_) during the KI-LMI task. The red and blue rectangles indicate the time (T^i^_no_ and T^i^_Kl_) it takes to reach E_min^i^_no_ for the LMI task. **(C)** and **(D)** Statistical test of E_min^i^_no_/E_min^i^_Kl_ and T^i^_no_/T^i^_Kl_. E_min_no_, E_min_KI_, T_no_ and T_KI_ represent the mean value of E_min^i^_no_, E_min^i^_Kl_, T^i^_no_ and T^i^_Kl_ for all subjects, respectively. Data: mean ± SD. **p < 0.01, *** p < 0.001.

The Cz-ERD power of LMI under 8-30 Hz is analyzed (see Figure 7), since the above topographical distribution displays a stronger feature around the Cz electrode. The Cz-ERD power of no-LMI shows a significant decreasing trend at the beginning of the task except for Subject H3, H5, H7 and H13(see Figure 7A). Especially, the ERD power of Subject H3, H7, and H13 shows a slight decreasing trend after adding KI to the LMI task (i.e., KI-LMI). For most subjects, the ERD tendency was more obvious and the lowest ERD power (i.e., E_min) was lower during the KI-LMI task. Figure 7B displays the average ERD of all subjects. These results manifest that the average ERD tendency is more obvious during the KI-LMI task. On the one hand, the E_min value is significantly smaller during the KI-LMI task than the no-LMI task ( $E\_\min_{\mathrm{KI}}$ < $E\_\min_{\mathrm{no}}$ , *p* < 0.001, see Figure 7D). On the other hand, $T_{\mathrm{KI}}$ is significantly shorter during the KI-LMI task than the no-LMI task ( $T_{\mathrm{KI}}$ < $T_{\mathrm{no}}$ , *p* < 0.01, see Figure 7C).

**Figure 8:**
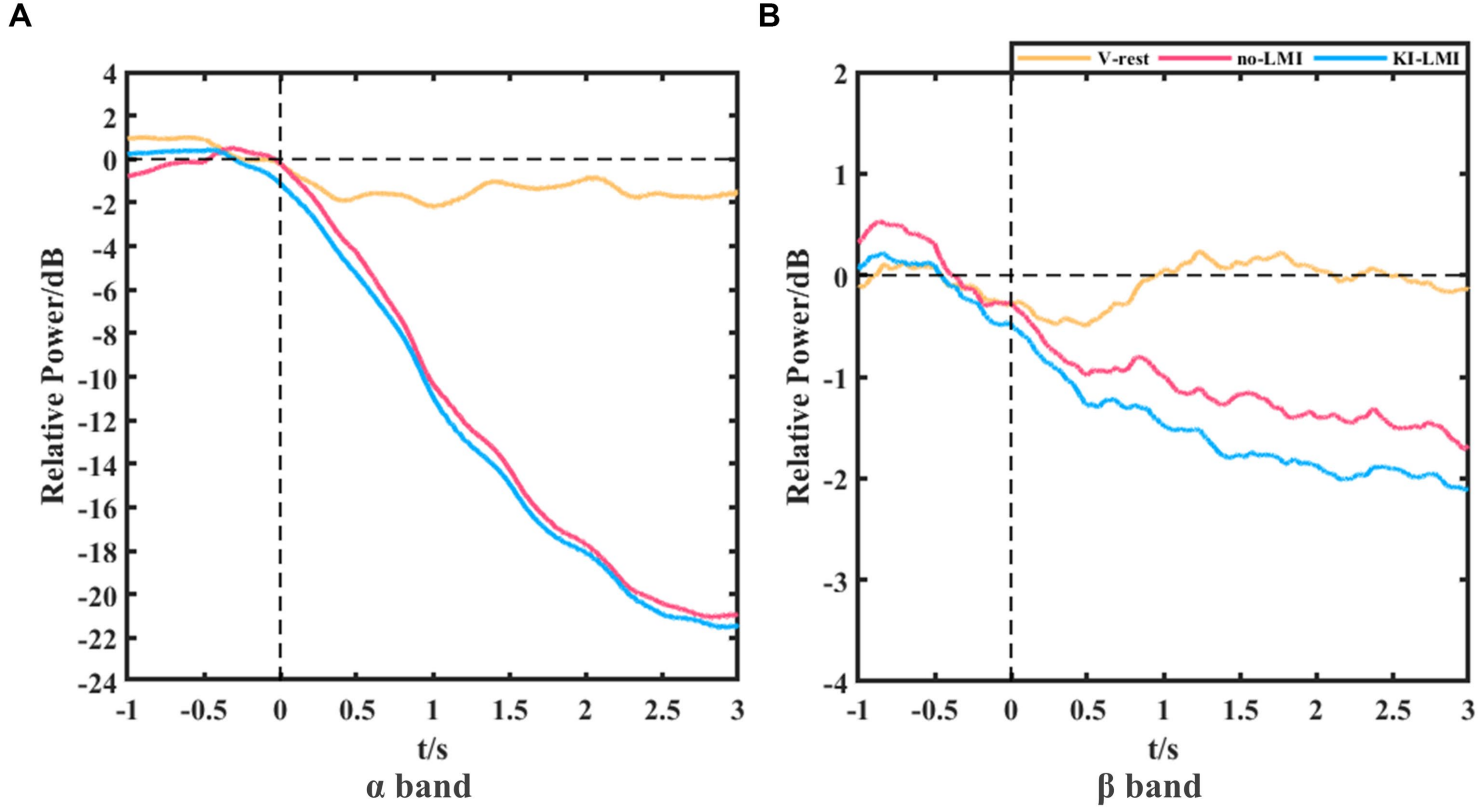
Grand-averaged relative power of Cz electrode for V-rest, no-LMI and KI-LMI task relative to the rest task at α band **(A)** and β band **(B)**.

Figure 8 shows the grand-averaged relative power of Cz electrode for V-rest, no-LMI and KI-LMI task relative to the rest task at α and β bands. The results in the α band included the following (see Figure 8A): (1) During the V-rest task, there was a slight decrease in relative power over the whole task period; (2) During the no-LMI task, there is a large relative power decline, and the peak value reaches -21 dB; (3) Compared with the no-LMI task, KI-LMI task produced a more significant relative power decline, with a peak value of −21.5 dB. The results in the β band included the following (see Figure 8B): (1) During the V-rest task, relative power decreased within 0 ~ 1 s and fluctuated around the baseline within 1 ~ 3 s; (2) Compared with the α band, the β band produced a similar power decline trend during the LMI, but had a smaller peak value (i.e., no-LMI: −1.7 dB, KI-LMI: −2.1 dB).

#### 3.2.2. Results of brain functional connectivity analysis for no-LMI/KI-LMI task

The iCOH of functional connectivity patterns was analyzed to plot the functional connectivity map and explore the effect of KI on brain activity. The functional connectivity map (see Figure 3) was grand-averaged across subjects for the two conditions with the alpha and beta frequency bands separately. In the Figure 3, The color variation and thickness of the connecting lines indicate the strength of functional connectivity between channels. During the no-LMI task, the channels had weak functional connectivity in the α band and strong functional connectivity in the β band. During KI-LMI, KI enhances the functional connectivity between the channels in both α and β bands. In particular, the contralateral cortical connectivity was enhanced, such as channels FC1, FC3, CP3, C1, C3, C5, Cz, etc. These results indicate that KI can improve the spatial feature distribution of the brain during performing LMI task, enhance the connectivity among cerebral cortical channels, and thus improve the information transmission process between brain regions.

#### 3.2.3. Classification performance of rest/V-rest and no-LMI/KI-LMI

**Figure 9:**
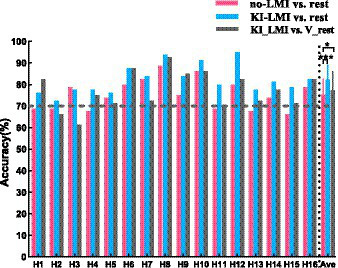
Comparison of classification accuracy across subjects. The black dashed line represents 70% of accuracy. Data: mean ± SD. * *p* < 0.05.

Figure 9 shows the comparison of classification accuracy across subjects for classifying LMI and rest task (i.e., no-LMI vs. rest and KI-LMI vs. rest). The subjects with less than 70% classification accuracy are defined as BCI-illiterate (Shu et al., 2017; Zhang et al., 2021). As shown in Figure 9, six healthy subjects (H1, H2, H4, H11, H13 and H15) were BCI-illiterate during the no-LMI task. After adding the KI to the LMI (i.e., KI-LMI), average accuracy of these five subjects increased by 9.17%. In addition, KI generally improves LMI-BCI offline accuracy and F1_score except for subject H3, and the accuracy and F1_score of all subjects were greater than 70%. Especially, four subjects (H6, H8, H10 and H12) reached a higher BCI accuracy (> 85%). Classification results were statistically evaluated using a paired t-test method. The offline accuracy was significantly improved for classifying LMI and rest (*p* < 0.001), achieving a 6.88% improvement and reaching 82.19%. And, the results of Table 3 indicates that average F1_score value increased by 7.6% after adding KI to LMI task (i.e., KI-LMI task). Seeing Table 3 for detailed F1_score of each participant.

**Table 3:** Results of the F1_score across subjects.

Task	Subject/F1_score (%)																
	H1	H2	H3	H4	H5	H6	H7	H8	H9	H10	H11	H12	H13	H14	H15	H16	Ave
Class1	63.9	66.8	**78.7**	65	75.0	80.5	83.0	87.7	70.8	86.7	65.1	80.2	66.9	74.1	64.9	79.5	74.3
Class2	76	**70.1**	74.9	**76.1**	**77.3**	87.9	**84.2**	**93.8**	82.1	**92**	**80**	**94.8**	**77.3**	**81.7**	**78.5**	**83.7**	**81.9**
Class3	**81**	65.5	58.3	71.1	69.1	**88.2**	72.3	92.3	**83.9**	86.1	69.3	81.4	72.9	79.1	68.9	83.5	76.4

Class1 represents the classification of rest and no-LMI; Class2 represents the classification of rest and KI-LMI; Class3 represents the classification of V-rest and KI-LMI; The bold number represents the maximum value for each subject.

Additionally, Figure 9 and Table 3 shows the results of classfing V-rest and KI-LMI. After adding vibration to the LMI and rest task (i.e., KI-LMI and V-rest), the simulated online average classification accuracy and F1_score of most subjects (H1, H4, H6, H8, H9, H11, H12, H13, H14, H15and H16) was improved (average accuracy: 79.84% > 74.09%, average F1_score: 79.24% > 72.6%), and there were significant differences in two groups (*p* < 0.05). And, the simulated online average performance for all subjects was presented in Figure 9 and Table 3 (i.e., average accuracy: 77.23 > 75.31%, and average F1_score: 76.4 > 74.3%).

#### 3.2.4. Oral questionnaire (Q2 and Q3)

After completing all the experimental tasks, each subject was given an oral questionnaire (Q2 and Q3) to explore the superiority of this research paradigm. Its results are shown in Table 2. Twelve subjects thought that KI was more conducive to focusing on LMI task. Thus they preferred the KI-LMI paradigm. In contrast, four subjects thought that a slight vibration is not enough to produce KI to enhance their LMI ability. Additionally, one subjects (H3) thought that a strong vibration distracted his attention on LMI.

#### 3.2.5. Statistical test of C3/Cz/C4-ERD power

The topographical distribution in Figure 5 shows more obvious contralateral cortex activation during right lower-limb MI task. In order to explore the activation effect of KI on different major channels, the paired T-test analyzed the $\bar{\mathrm{ERD}}$ power of channels C3, Cz and C4 under the no-LMI/KI-LMI tasks (see Table 4). For the channels C3 and Cz, there are significant differences (*p* = 0.006 < 0.01 and *p* = 0.007 < 0.01) in α frequency band, and there are significant differences (*p* = 0.001 and *p* = 0.000 < 0.001) in α + β frequency band. There are significant differences (*p* = 0.026 < 0.05) in β frequency band at channel C3. Nevertheless, for the channel C4, all *p*-values are far bigger than 0.05, which indicates there is no significant difference.

**Table 4:** Statistical results of the paired *t*-test.

Channel	*P*-value of no-LMI vs. KI-LMI		
	α (8-13 Hz)	β (14-30 Hz)	α + β (8-30 Hz)
C3	0.006**	0.026*	0.001***
Cz	0.007**	0.094	0.000***
C4	0.866	0.148	0.191

This table compares the $\bar{\mathrm{ERD}}$ power of two tasks in the same condition. Data: mean ± SD. *p < 0.05, **p < 0.01, ***p < 0.001.

## 4. Discussion

In the previous research, it was well known that brain injury often inhibits the LMI-ability after stroke, making the detection of EEG features harder (Takeda et al., 2007; Meyer et al., 2016; Park et al., 2016). The Higher BCI performance generally accelerates the patients’ recovery process (Guan, 2016). Therefore, enhancing LMI ability of stroke patients is critical to achieve rehabilitation. The latest study revealed that mimicking known biological control principles could improve BCI performance for subjects (Flesher et al., 2021). A natural way of interaction is acceptable to users, which increases its usefulness in practice (Xu et al., 2021). Previous studies used visual induction consistent with motor tasks to enhance LMI ability (Boord et al., 2010, Kitahara et al., 2017, Yu et al., 2018). Additionally, kinesthetic illusion (KI) is a type of proprioception that can complement the biological control loop. Two studies verified that a combination of upper-limb MI and KI feedback improved MI-BCI performance (Barsotti et al., 2018; Song and Kim, 2019). However, no previous study has investigated enhancing LMI ability by KI to design LMI-BCI paradigm. Therefore, based on the above researches, we designed two research contents to verify the feasibility of inducing KI *via* vibrating Achilles tendon, analyze the EEG features produced by KI, and explore whether KI could enhance LMI ability.

Long-term research found that KI, where one feels muscle stretch, could be induced by artificially vibrating the muscle spindle and tendon of the limbs (Naito, 2004; Tapin et al., 2021). Neuroimaging studies have revealed that KI and MI have similar activation patterns in brain regions (Naito and Ehrsson, 2001; Naito, 2004; Giannopulu and Mizutani, 2021). In addition, vibration is better transmitted to the muscle spindle if applied over the tendon, where it results in longitudinal stretch of the muscle fibers (Taylor et al., 2017). Vibration stimulation of tendon is an effective way to induce KI (Barsotti et al., 2018; Tapin et al., 2021). In this study, the most obvious tendon location (i.e., Achilles tendon) was selected as the vibration stimulation point. In medical contexts, using a MI questionnaire as an ability predictor tool could be one possible way to estimate BCI performance (Vasilyev et al., 2017; Rimbert et al., 2018). In the field of BCI, two classical work studied two different MI questionnaires. The first study concludes that the KMI scores obtained from the Kinesthetic and Visual Imagery Questionnaire could predict the performance of a MI-based BCI for able-bodied subjects (Vuckovic and Osuagwu, 2013). The second study found that the representation of subjective behaviors, calculated using the Motor Imagery Questionnaire Revised-Second Edition, and the control of the BCI seem to be strongly linked (Marchesotti et al., 2016). These studies have shown that MI questionnaires are probably the most accepted and validated methods to measure the subjective feelings of a subject. Therefore, our study conducted an oral questionnaire for all subjects to explore the results of the experiment. In this study, firstly, an oral questionnaire (Q1) of Table 2 verified that KI was felt subjectively by most subjects (13 out of 16 subjects) during vibrating Achilles tendon. Secondly, KI induced by vibratory stimulation enhanced the activation of the area around the Cz and CPz electrodes (i.e., around the sensorimotor cortex) during the V-rest task (see Figure 5). This result is consistent with previous research that activated cortex area (i.e., contralateral primary motor cortex) by KI was similar to the activated cortex area by MI (Rosenkranz and Rothwell, 2003; De Moraes Silva et al., 2015; Lopez et al., 2017; Giannopulu and Mizutani, 2021; Zhang et al., 2021). Similarly, a recent study showed that vibrotactile neurofeedback training on upper limbs can increase motor cortical excitability in hand muscle representation corresponding to a muscle engaged by the MI (Grigorev et al., 2021). Lastly, the classification performance of rest and V-rest shows a significant difference (see Figure 6), which suggests that activated the independent effects on the cerebral cortex by KI can be classified clearly. This experimental study verified that induced KI *via* vibrating Achilles tendon may be feasible, and provided a theoretical basis for applying KI to LMI-BCI paradigm. In order to better explore the difference and correlation between LMI and KI, we need to ensure the same duration of LMI and KI, so this study chose the same induction duration as LMI (i.e., 3.5 s). The above results verified the effectiveness of KI induction with a duration of 3.5 s. However, there are still some defects in this study. 3.5 s may not be the best KI induction duration. Future studies will investigate the optimal duration of KI induction.

Both studies demonstrated that a combination of upper-limb MI and KI feedback improved MI-BCI performance (Barsotti et al., 2018; Song and Kim, 2019). In our study, we used many analytical methods (e.g., ERD power, topographical distribution, oral questionnaire, and functional connectivity analysis) to explore whether KI enhances LMI ability. Previous studies explored functional connectivity of different brain regions based on Granger causality analysis. These studies found that the significant causal connection from the visual area to the motor area under the “visual–auditory context” and the “visual context” may indicate the information transmission process of the dorsal pathway evoked by the visual stimulus. Therefore, our study used iCOH algorithm to analyze the brain functional connectivity between different channels. The results in Figure 3 indicate that KI can enhance the information transmission process between brain regions (especially the sensorimotor area) during performing the LMI task, thus improving LMI ability. In previous mechanism studies, the anatomical location of the lower limbs motor cortex deep within the central cortex of the interhemispheric fissure, which corresponds to the Cz electrode of the 10/10 standard electrode distribution system (Neuper and Pfurtscheller, 1996; Jurcak et al., 2007; Boord et al., 2010). Therefore, in our study about LMI, the Cz electrode was selected to analyze the ERD trend in the 8–30 Hz band during LMI tasks. When adding KI to the LMI task (i.e., KI-LMI), the cortex activation around the Cz electrode is more significant (see Figure 5), and the Cz-ERD tendency was more obvious for most subjects (see Figure 7A). To evaluate the MI enhancements of the proposed paradigm, we compared the observed ERD for different LMI tasks. The peak ERD amplitudes (i.e., $E\_\min_{\mathrm{no}/\mathrm{KI}}^{i}$ ) were used to quantitatively evaluate the enhancement due to more immersive LMI from the paradigms (Song and Kim, 2019). Moreover, the ERD arrival time (i.e., $T_{\mathrm{no}/\mathrm{KI}}^{i}$ ) was adopted as an indicator of temporal characteristics of ERD because it was used to evaluate the appropriateness of ERD for BCI, which is also an MI enhancement target for BCI system (Duann and Chiou, 2016; Song and Kim, 2019). In our study, $T_{\mathrm{KI}}$ is significantly shorter during the KI-LMI task than the no-LMI task ( $T_{\mathrm{KI}}$ < $T_{\mathrm{no}}$ , see Figure 7C). Therefore, the ERD feature of KI-LMI was detected more quickly (i.e., better real-time; Duann and Chiou, 2016; Song and Kim, 2019), then the KI may speed up the online detection of LMI-BCI. The above significant ERD indexes are beneficial to EEG feature detection, thus improving the classification performance of BCI system. Referring to previous research methods (Storzer et al., 2016), this study plotted the grand-averaged relative power of Cz electrode (see Figure 8), in order to more clearly compare the effect of KI on different tasks. The results of Figure 8 and Figure 5 are consistent, and both show that relative power in the α band presents a more significant decline trend than that in the β band. This result indicated that the optimal frequency band of performing LMI is 8-13 Hz for some subjects in this study. And, above results suggest that KI enhances the relative power decline for all frequency bands during performing LMI and improves LMI ability of subjects.

In terms of BCI performance, KI significantly improved the average accuracy of classifying LMI and rest task (see Figure 9), achieving a 6.88% improvement. Especially, it is noteworthy that the average accuracy of all BCI-illiterate increased by 9.17%. Therefore, KI could enhance LMI, and enhanced LMI by KI may be more suitable for the BCI-illiterate (e.g., the partial stroke patients; Shu et al., 2017, 2019; Ren et al., 2020). Considering that if KI is applied to online LMI-BCI, vibration stimulation need to be performed simultaneously during LMI and rest tasks. Therefore, KI-LMI and V-rest are classified in our study to simulated online BCI performance. The results of this study show that KI is more conducive to the online classification of LMI and rest tasks for most subjects(see Figure 9, KI-LMI vs. V-rest, *p* < 0.05). Grand-averaged topographical distribution indicates that EEG features of KI-LMI and V-rest tasks have significant differences (i.e., More significant ERD feature were observed under the KI-LMI task). Therefore, better results can be obtained by classifying KI-LMI and V-rest tasks. Obviously, the high classification performance of KI-LMI/V-rest tasks is more suitable for the practical application of LMI-BCI paradigm. In addition, the results of Figure 7 and Figure 8 show that KI can enhance ERD feature and relative energy changes for most subjects. This result is more helpful for CSP to extract the two types of features with significant differences, so as to improve the classification performance (see Figure 9). In contrast, one subject (H3) thought that KI distracted his attention during the LMI task. Our research analyzed that higher vibration frequency might make this subject nervous because the Cz-ERD power of this subject was relatively unstable before the LMI task (see Figure 7A). The offline accuracy of the H3 is decreased by 1.25% (see Figure 9) due to the above reasons. Our research paradigm presented significant performance advantages for most subjects. Consequently, it may be more conducive to the training and rehabilitation of most stroke patients. Previous studies have found that activated areas of the cerebral cortex are biased toward the contralateral sensorimotor cortex during performing the LMI and visual observation task (Li et al., 2015; Yu et al., 2018). In this research (Tariq et al., 2020), LDA, SVM, and KNN model were used to classify bilateral foot LMI task. And, the single trial analysis and classification models resulted in high discrimination accuracies, i.e., maximum 83.4% for beta-ERS, 79.1% for beta-ERD, and 74.0% for mu-ERD. The above results are consistent with our study in Figure 5. Additionally, our statistical results in Table 4 proved that the KI significantly enhanced the cortex activation at the left and middle region of sensorimotor cortex (i.e., channels C3 and Cz) during the LMI task. This result is consistent with the topographical distribution (see Figure 5). Therefore, the KI may be conducive to distinguishing the left/right LMI task. This study provides a new idea to design the left/right LMI-BCI paradigm.

Previous studies have shown that 70 to 80 Hz is an effective way to induce KI, and high vibration frequencies cannot induce KI (Naito et al., 1999; Naito, 2004; Taylor et al., 2017). The results of our study indicate that 180 Hz can induce lower-limb KI, which is somewhat different from previous results. Different vibration stimulation locations will induce different KI effects. Previous studies explored the optimal stimulation frequency range through vibration of the upper limb tendon (Naito et al., 1999; Naito, 2004; Taylor et al., 2017; Barsotti et al., 2018), while this study explored the effect of inducing lower-limb KI through vibration of Achilles tendon. The cerebral cortex region corresponding to the upper-limb and lower-limb has a great difference in physiological structure (i.e., the anatomical location of the lower-limb motor cortical area is smaller and deep within the contralateral mesial cortex; the lower limb is located at the distal physical level) (Michael, 2016), which makes the optimal vibration frequency for inducing the upper-limb and lower-limb KI may have great difference. The experimental results of our study verified the feasibility of lower-limb KI induced by vibration of Achilles tendon at 180 Hz, which provides some reference for the research on inducing lower-limb KI with vibration stimulation. However, the vibration parameters (e.g., vibration frequency and vibration duration) were not optimized in this study. In the future, the influence of different vibration parameters on inducing lower-limb KI will be explored to produce the optimal induction effect. Studies have shown that MI-BCI is beneficial to improve the rehabilitation of stroke patients (Mane et al., 2020). Therefore, our research paradigm further improves the rehabilitation effect and lower-limb motor function of stroke patients by improving the performance of LMI-BCI. However, our study removed data with more artifacts before classification, which may affect the results of online classification in LMI-BCI. Future research will design online experiments to explore the effect of removing data with more artifacts on online application. Although the obtained results are statistically significant, it is acknowledged that the sample size of the current study is limited to 16 healthy subjects. Therefore, more healthy subjects and stroke patients should be recruited to further study the features in sensorimotor cortex caused by KI in different groups.

## 5. Conclusion

In this study, we integrated the induced KI by vibrating Achilles tendon with LMI tasks to enhance LMI ability. Our research 1 has verified that the KI was induced by vibratory stimulation on Achilles tendon, and found that the activated cortex area by the KI was similar to the activated cortex area by LMI. Research 2 demonstrated that the KI could enhance the activation of the sensorimotor cortex and improve the classification preformance of offline and simulated online LMI-BCI. Additionally, this study found that KI improved the ipsilateral difference of cerebral cortex. The LMI-BCI paradigm of this study is conducive to enhance LMI ability and provides a new idea to design a novel left/right LMI-BCI paradigm. This proposed approach enriched the content of LMI-BCI technology and accelerated its research progress.

## Data availability statement

The raw data supporting the conclusions of this article will be made available by the authors, without undue reservation.

## Ethics statement

The studies involving human participants were reviewed and approved by the Ethics Committee of Xi’an Jiaotong University (Approval No. 2021–1577). The participants provided their written informed consent to participate in this study.

## Author contributions

WW, BS, and JW contributed to conception and design of this study. WW and DW carried out collecting EEG data and statistical analysis. BS, GL, and JW provided many suggestions for this study. WW and GL worked together to complete the manuscript. All authors contributed to manuscript revision, read and approved the submitted version.

## Funding

This work was supported by the open project of Henan Key Laboratory of Brain Science and Brain-Computer Interface Technology under Grant HNBBL230101 and HNBBL230207, and the Shenzhen Science and Technology Plan projects under Grant JSGG20201102145602006, and 2021 Basic Research Project of Shenzhen Science, Technology and Innovation Commission under Grant JCYJ20210324123414039.

## Conflict of interest

The authors declare that the research was conducted in the absence of any commercial or financial relationships that could be construed as a potential conflict of interest.

## Publisher’s note

All claims expressed in this article are solely those of the authors and do not necessarily represent those of their affiliated organizations, or those of the publisher, the editors and the reviewers. Any product that may be evaluated in this article, or claim that may be made by its manufacturer, is not guaranteed or endorsed by the publisher.
